# A Review of Reviews Assessing Patient–Provider Racial and Ethnic Concordance in Mental Health

**DOI:** 10.1007/s40615-025-02546-y

**Published:** 2025-07-21

**Authors:** Jessica L. A. Jackson, Kyle Patrick Apilado, Tracey Pérez Koehlmoos

**Affiliations:** 1 Department of Preventive Medicine & Biostatistics, Uniformed Services University for the Health Sciences, 4301 Jones Bridge Road, Bethesda, MD 20814 USA; 2 Henry M. Jackson Foundation for the Advancement of Military Medicine, Inc, 6720 A Rockledge Dr, Bethesda, MD 20817 USA; 3 Center for Health Services Research, Uniformed Services University for the Health Sciences, 4301 Jones Bridge Road, Bethesda, MD 20814 USA

**Keywords:** Systematic review, Physician–patient racial and ethnic concordance, Mental health

## Abstract

**Introduction:**

Physician–patient racial and ethnic concordance is an oft studied area within the field of mental health. As research on this topic has increased in volume and complexity over the years, it is worth examining the lessons learned. The objective of this paper was to assess reviews related to this topic to find commonalities in the literature and recommend future steps for research.

**Methods:**

A review of systematic reviews was conducted to rapidly summarize the evidence related to physician–patient racial and ethnic concordance in mental health. A comprehensive search was run in PubMed, EMBASE, PsychINFO, and Web of Science databases. Covidence was used to manage the review process from title screening to extraction. The AMSTAR-2 tool was used to assess the quality of the included systematic reviews.

**Results:**

Ten reviews were included in this study. While the collective results of the reviews were heterogenous, there were commonalities identified. For example, Black clients typically experience poorer mental health outcomes in comparison to their counterparts and racial/ethnic matching tends to have a greater effect for this group. Additionally, a few articles discussed the importance of understanding and integrating cultural perspectives into therapeutic techniques and interventions.

**Discussion:**

There is some evidence that racial and ethnic concordance for certain groups can assist in building rapport in the therapist-patient relationship. Future research should focus on multiple measures of concordance, the intersection between them, and investigate mediators and moderators to therapeutic outcomes. Furthermore, cultural considerations should be meaningfully incorporated into every aspect of mental health service.

## Introduction

Physician–patient racial and ethnic concordance is an oft studied area within the field of mental health, with research dating back to at least 1963 [1]. The impetus for this research began with a recognition of differential mental health outcomes for Black patients among the therapist community [2]. Then, and now, the debate centered on reasons for ineffective treatment and solutions. Racial/ethnic concordance was explored as a way to provide patients with socio-cultural competent providers in order to enhance treatment outcomes [2]. However, the research has largely yielded inconclusive results with some affirming the utility of concordance for specific groups, while others have found no differences in outcomes [3, 4].

For example, a study conducted by Takeuchi, Sue, and Yeh on the return rates and outcomes from ethnicity-specific mental health programs found that patients who were in ethnically matched mental health programs had higher return rates and treatment time in comparison to those in mainstream programs [5]. Another study conducted by Mujica et al. studied the relationship between patient-centered communication and racial and ethnic concordance in outpatient mental health clinics [6]. The authors found no significant differences were found between racially and ethnically concordant or discordant dyad in terms of incorporating patient-centered communication in therapy sessions with clients [6].

As research on this topic has increased in volume and complexity over the years, it is worth examining the collective lessons learned. Many authors have conducted reviews related to racial and ethnic concordance in healthcare from various lenses, including the utility of culturally adapted services [7–11] as well as barriers and facilitators for specific groups [12–15]. However, there are few systematic reviews specific to the effect of racial and ethnic concordance within the field of mental health. To that end, the objective of this paper was to assess reviews related to physician–patient racial and ethnic concordance in mental health in order to find commonalities in the literature and recommend future steps for research.

## Methods

The overarching goal of this paper is to understand the current state of research related to physician–patient racial and ethnic concordance within the field of mental health. One way to achieve this is to perform a review of systematic reviews. Systematic reviews are useful for synthesizing existing literature in order to improve precision, accuracy, and confidence in findings related to a specific topic [16]. As such, they are often used to inform evidence-based decision-making in medicine [17]. A review of systematic reviews is used to rapidly summarize the evidence from multiple systematic reviews. Additionally, they are used to compare and contrast the findings from existing reviews to provide a comprehensive examination into a particular topic [18].

### Inclusion and Exclusion Criteria

Inclusion criteria for this study was defined along the dimensions of population, intervention or outcome of interest, comparison/control group(s), and context (PICO). The population of interest were reviews related to physician–patient racial/ethnic concordance within the field of mental health. No age group was specified so as to be inclusive of all services and types of mental health providers. The interventions of interest for this study encompassed all types of mental health services offered along the care continuum, including services offered in public health, community, rural, medical, and academic settings. The comparison of interest was historically marginalized or minoritized groups in contrast to their counterparts. Outcomes of interest for within selected reviews included factors such as access to treatment, retention, and alleviation of symptoms for a variety of mental illnesses. Finally, only reviews published in English and after the year 2000 were included in this review. Articles were limited to English due to the team’s language proficiency and to after the year 2000 to capture articles with a greater probability of using a critical appraisal tool. Inclusion was open to articles in peer-reviewed academic journals, theses, and dissertations.

Exclusion criteria for this study comprised articles published in a language other than English, and published before the year 2000. Furthermore, publications were excluded if they did not conduct a systematic review or meta-analysis, were in a field of medicine other than behavioral health, or focused on comparisons of populations that did not include historically marginalized or minoritized populations.

### Literature Search

The team performed a search of the published literature on November 21, 2023, and again on July 30, 2024, to ensure thoroughness. The search was conducted using PubMed, EMBASE, PsychINFO, and Web of Science databases. The search strategy included a title and abstract search databases using keywords, root terms, and synonyms related to race and ethnicity, concordance, matching, therapist, counselor, psychotherapist, psychologist, clinician, social worker, nurse practitioner, general, family practitioners, systematic review, and meta-analysis. Table 1 has the complete list of search terms, keywords, and operators.

**Table 1:** Search strategy and keywords

PubMed	(("Psychiatry and Psychology Category"[mh] OR"Nurse Practitioners"[mh] OR"Social Workers"[mh] OR therapist*[tiab] OR psychologist*[tiab] OR psychotherapist*[tiab] OR clinician*[tiab] OR social-work*[tiab] OR nurse-practioner*[tiab] OR psychiatrist*[tiab]) AND (“race concordance"[tiab: ~ 2] OR"racial concordance"[tiab: ~ 2] OR"ethnic concordance"[tiab: ~ 2] OR"race match"[tiab: ~ 2] OR"race matching"[tiab: ~ 2] OR"racial matching"[tiab: ~ 2] OR"racial match"[tiab: ~ 2])) AND ((meta-analysis[Filter] OR systematicreview[Filter]) AND (2003:2024[pdat]))
PsychInfo	((“Racial and Ethnic Groups” OR race OR racial OR ethnic*) AND (concord* OR match*)) AND (therapist or counselor or psychotherapist or psychologist or clinician or social worker* OR nurse practitioner*) Filters: 2003–2024, Systematic Reviews and Meta-Analyses
Embase	('psychology'/exp OR'psychology'OR'psychiatry'/exp OR'psychiatry'OR'psychiatric treatment'/exp OR'psychiatric treatment'OR'nurse practitioner'/exp OR'nurse practitioner'OR'social worker'/exp OR'social worker'OR'psychotherapist'/exp OR'psychotherapist'OR'therapist'/exp OR'therapist'OR'psychologist'/exp OR'psychologist'OR'psychiatrist'/exp OR'psychiatrist'OR'clinician'/exp OR'clinician') AND ('racial concordance'OR'race concordance'OR'race match*') Filters: 2003–2024, Systematic Reviews and Meta-Analyses
Web of Science	TS = ("race matching"OR"racial matching"OR"race match*"OR"racial concordance"OR"race concordance"OR"ethnic concordance") AND TS = (psychology OR psychiatry OR therapist OR psychotherapist OR"nurse practitioner"OR"social work*"OR psychologist OR psychiatrist OR clinician) and 2023 or 2022 or 2021 or 2020 or 2019 or 2018 or 2016 or 2008 or 2010 or 2009 or 2011 or 2013 or 2014 or 2015 or 2007 or 2006 or 2005 or 2004 or 2003 (Publication Years) and"Systematic Review"Or"Meta-analysis"(Search within all fields)

### Study Selection

Each step of the selection and extraction process was performed either in triplicate or duplicate. First, three reviewers used Covidence to screen each title and abstract. Two reviewers (JLAJ and KA) then assessed full-text articles and performed data extractions. Conflicts as to the relevance of articles for exclusion were settled by a third reviewer (TK). From each included review, the following information was extracted: authors, number of included studies, country, study characteristics (type of review, aim of study, inclusion and exclusion criteria, and outcomes of interest), and key findings. Meta-analysis was not conducted due to the heterogeneity of the included reviews.

### Critical Appraisal

Critical appraisal is the hallmark of a high-quality systematic review. Based on the Cochrane Collaboration Handbook, AMSTAR 2 allows for an assessment of randomized and non-randomized interventions thus enhancing its utility [19, 20]. After the full-text review, two reviewers (JLAJ and KA) independently applied this tool to each study to assess the quality of the work and rate the overall confidence in the key findings. Conflicts as to the quality of the articles were settled by a third reviewer (TK).

## Results

### Search Results

**Fig. 1:**
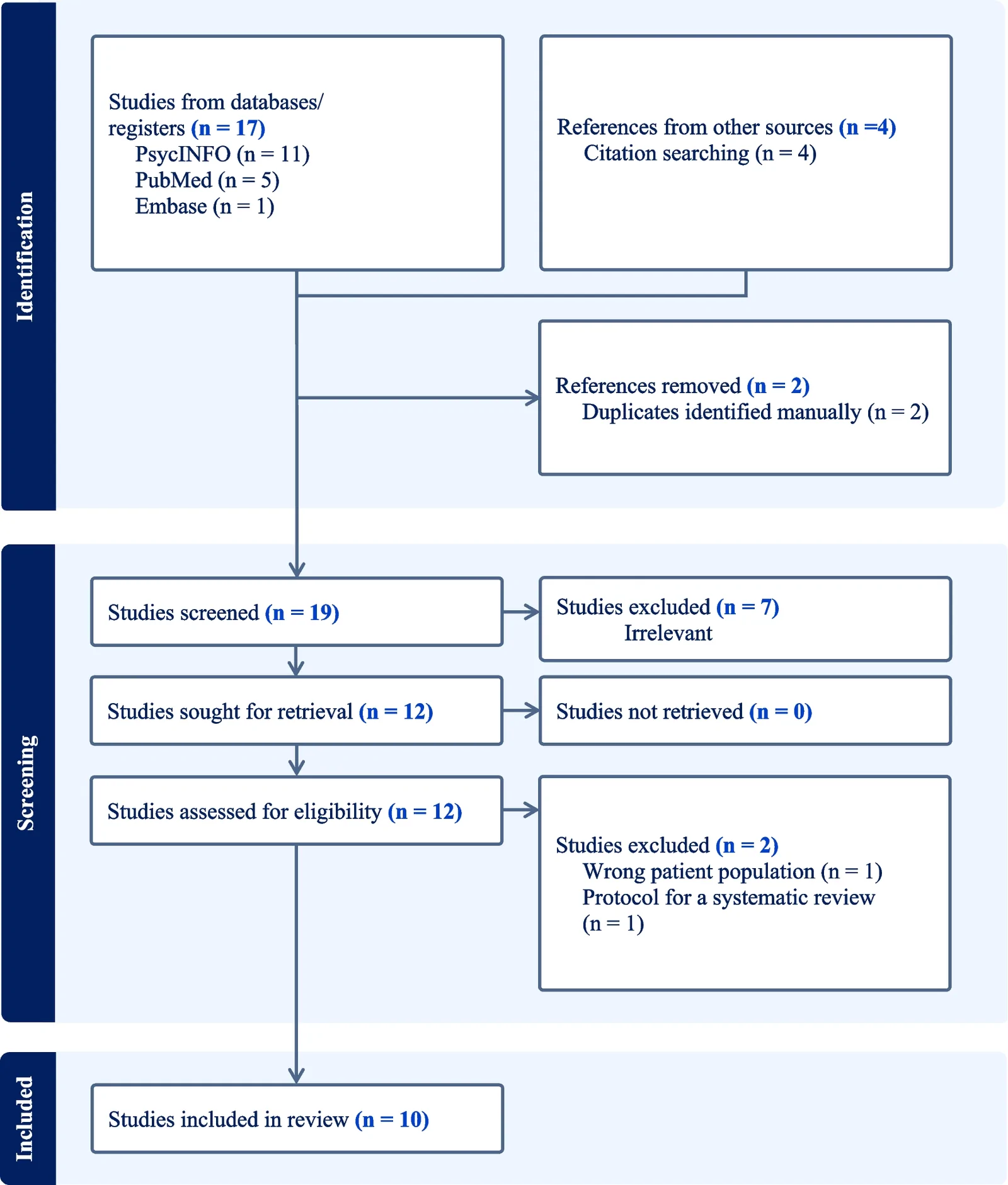
PRISMA flowchart

An initial search of databases identified 17 abstracts that met the criteria for this study. Four additional reviews were identified by citation searching for a total of 21. Two duplicates were removed, and seven failed to meet inclusion criteria during the title and abstract review [21–27]. A total of 12 reviews were examined during the full-text review, and two were eliminated. One article was a protocol for a future systematic review and did not contain the actual study [28]. The other publication focused on the global mental health experiences of single mothers without a focus on race and ethnicity. Thus, this study was excluded due to a focus on an ineligible population [29]. Ten publications were included in the final synthesis. The process for selecting and excluding reviews is shown in a PRISMA flowchart in Fig. 1.

### Characteristics of Reviews

A description of reviews included in this review is provided in Table 2. The included reviews were published between 2001 and 2024. The most frequent study design was meta-analyses (*n* = 5) followed by systematic reviews (*n* = 4), along with one scoping review. The systematic reviews followed a specified process to comprehensively search, assess, appraise, and summarize research related to physician–patient ethnic and racial concordance. In comparison, the meta-analyses started with a systematic review and used statistical methods to pool the results from numerous studies in order to provide a more precise estimate of the effect size. All of the publications included non-randomized studies of interventions in their review, with no randomized control trials. The total number of studies included within each review ranged from 7 to 76, and the average number of studies included in each review was 42. Twenty-six studies were shared between two systematic reviews, while two studies were shared between three or more systematic reviews. All other studies were unique to their respective reviews. Table 3 displays the list of publications that overlap between reviews. Lastly, seven reviews included populations solely based in the USA [9, 30–35], two included populations in North America and Europe [36, 37], and one was exclusive to European populations [38].

**Table 2:** Description of included studies

Authors	Title	Corresponding author contact details	Country	Number of studies	Aim of study	Study design	Inclusion criteria	Exclusion criteria	Outcome of interest	Key findings
Cabral and Smith (2011)	Racial/ethnic matching of clients and therapists in mental health services: a meta-analytic review of preferences, perceptions, and outcomes	Timothy B. Smith; tbs@byu.edu	USA	154	To summarize relevant research related to (a) patients’ preferences for racial/ethnically concordant therapists, (b) perceptions of therapists across racial/ethnic pairings, and (c) impacts of racial/ethnic concordance on therapeutic outcomes	Meta-analysis	1.) Provided quantitative data regarding individuals’ preferences, perceptions, or outcomes relevant to racial/ethnic matching with mental health therapists	1.) Case studies, 2.) Single-subject designs, and 3.) Qualitative research	1.) Patients’ preferences for racial/ethnically concordant therapists; 2.) Perceptions of therapists across racial/ethnic pairings; and 3.) Impacts of racial/ethnic concordance on therapeutic outcomes	Mental health treatment outcomes do not substantively differ when clients do or do not have a therapist of their same race and ethnicity. Except in studies involving Black participants where effect sizes were the highest across all outcomes of interest. Overall, client race and ethnicity do moderate preferences, perceptions, and outcomes particularly for people of color than for White/European Americans
Carballeira-Carrera et al. (2020)	Clinical approaches to cultural diversity in mental health care and specificities of French transcultural consultations: a scoping review	Laura Carballeira Carrera; laura.carballeira@ salud.madrid.org	USA, UK, Canada, Australia, New Zealand, Sweden, Norway, Denmark, Italy, Germany, Spain, Netherlands, Belgium	29	To list and describe the multiple ways of providing psychiatric healthcare that is adapted to cultural diversity and compare these models to the French Transcultural Psychotherapy model	Scoping review	1.) Peer reviewed journal articles 2.) Dealing specifically with clinical models for handling cultural diversity in psychiatric treatment 3.) Referring to psychiatric healthcare services that provide care adapted to cultural diversity 4.) Describing the modalities of care 5.) Published between January 1, 1990, and October 1, 2018 6.) In English, French, or Spanish	1.) Articles which only referred to transcultural psychiatry at a theoretical level	The range and methods of adapting psychiatric care to a context of cultural diversity and a comparison of these methods to the French Transcultural Psychotherapy model	The major types of adaptations to psychotherapy include: innovations to the therapeutic framework such as ethnic matching, developing specialized services for sub-populations, and cultural competency training. However, the French transcultural psychotherapy model differs in that it emphasizes an integration of culture into the therapeutic model, rather than a feature of it. The authors conclude the objective is to change the paradigm to allow for cultural theories to inform symptoms, treatment options, and content for sessions
de Haan et al. (2018)	A review of mental health treatment dropout by ethnic minority youth	Anna M. de Haan; a.dehaan@ pharos.nl	USA, UK, Netherlands	27	To synthesize the findings of research on child and adolescent mental health therapy dropout rates and dropout predictors by ethnic minority group	Systematic review	1.) Peer-reviewed articles in English; 2.) Outpatient settings; 3.) Focus on psychiatric treatment in mental health services; 4.) Participants under 21 years of age; 5.) Patients whose ethnic background was described; and 6.) At least one minority ethnic group in the country where the study was performed	1.) Related to preventing recidivism; 2.) Related to medication management exclusively; 3.) Theoretical or qualitative; 4.) Focused on child retention or the number of visits without defining the status of termination; 5.) Participants were mandated to treatment; and 6.) Described patient ethnic backgrounds but did not discuss in the results or discussion	1.) Ethnicity-specific dropout predictors; and 2.) Ethnicity-specific dropout rates	The results revealed varying dropout rates by race and ethnicity, specifically for Hispanic or Mexican Americans, Asian/Asian Americans, and Dutch ethnic minorities compared to Whites. The only consistent finding was higher dropout rates for Black patients compared to Whites and other ethnic minorities Additionally, the review indicated that therapist-patient ethnic match decreased the likelihood of dropout, especially for adolescents; socioeconomic status was not a consistent predictor for dropout, regardless of ethnicity; and the quality of therapeutic relationship between therapists and patients may be a strong predictor of dropout, for all ethnic groups
Griner and Smith (2006)	Culturally adapted mental health interventions: a meta-analytic review	Timothy B. Smith; TBS@byu.edu	USA	76	To gather and synthesize the empirical literature of outcomes associated with culturally adapted mental health interventions using meta-analytic methodology	Meta-analysis	1.) Written in English 2.) Provided quantitative data evaluating a culturally adapted mental health intervention 3.) Manuscript explicitly states that e adaptations were based on culture, ethnicity, or race	1.) Studies that were adapted for other characteristics without regard for culture, ethnicity, or race 2.) Studies that assigned clients to therapists of the same ethnic group or native language without culturally appropriate adaptations to the content, format, or delivery of the intervention	1.) Client characteristics; 2.) Research characteristics; 3.) Types of cultural adaptations to the interventions; and 4.) Which types of outcomes were associated with the greatest benefits to clients	The authors found a moderately strong effect for culturally adapted behavioral health interventions. Specifically, interventions with a targeted cultural group were four times more effective than those open to a variety of cultural groups; and interventions offered in a clients'native language were twice as effective as those conducted in English
Maramba et al. (2002)	Meta-analyses of ethnic match as a predictor of dropout, utilization, and level of functioning	Gloria Gia Maramba; jm150@psu.edu	USA	7	To summarize the overall effect of ethnic matching in psychotherapy as a predictor of treatment dropout, service utilization, and level of functioning	Meta-analysis	1.) Ethnic matched psychotherapy study; 2.) Predicted dropout, utilization, or global assessment score at termination of therapy	None listed	The strength of the relationship between ethnic matching and client dropout, service utilization, and level of functioning	Small effect sizes were found between number of sessions attended, client dropout, and service utilization. Overall, the review indicated ethnic match is not a significant clinical predictor of decreasing dropout after the first session or increasing number of sessions attended
Miller et al. (2024)	The relationship of race/ethnicity concordance to physician–patient communication: a mixed-methods systematic review	Ann Neville Miller; ann.miller@ ucf.edu	USA	33	To investigate a) How communication patterns differ in race and ethnicity concordant versus discordant physician–patient medical encounters; b) What explanatory variables have been associated with those differences; c) What patient outcomes have been associated with these differences	Systematic review	1.) Studies that include a comparison of concordant and discordant physician–patient encounters; 2.) All medical specialties; 3.) Practicing physicians (including residents); 4.) Investigates at least one communication-related variable in the encounter; 5.) Any clinical setting; 6.) Quantitative, qualitative, and mixed methods studies 7.) Published in English 8.) Published from 2006 to 2022	1.) Studies that only look at language concordance without consideration of ethnic, racial, or cultural concordance	(1) Differences in communication patterns ethnically, racially, and culturally concordant versus discordant patient-provider encounters; (2) Explanatory variables associated with differences in communication patterns; and (3) Patient outcomes that are associated with communication differences	In most analyses, after accounting for covariates, no relationship was found between race and ethnicity concordance and communication variables. Race and ethnicity concordance with their physician do not appear to influence the quality of communication for most patients from minoritized groups
Sánchez-Bahíllo et al. (2014)	Therapist characteristics that predict the outcome of multipatient psychotherapy: systematic review of empirical studies	Angel Sanchez-Bahillo; angelsanchezbahillo@hotmail.com	Spain, UK	17	(a) Undertake the first systematic review of the available literature on the therapist characteristics which can predict the outcome of multipatient psychotherapy, (b) Establish which therapist characteristics affect the outcome of multipatient psychotherapy and (c) Evaluate the quality of the empirical research on the impact of the characteristics of psychotherapists on the outcome of multipatient psychotherapy	Systematic review	1.) Primary and empirical research studies; 2.) Published in English, Spanish, or French; 3.) Based on multipatient psychotherapies; 4.) Studies with non-clinical populations; 5.) Studies that describe therapist characteristics prior to therapy	1.) Studies with therapeutic interventions like yoga or animal assisted interventions 2.) Studies focused on the impact institutional factors 3.) Qualitative studies	1.) Quantitative measures after the therapy, including patients'treatment satisfaction 2.) Retention in treatment	Ethnic matching improves the outcome of family therapy with drug abusing adolescents in some ethnic minorities and that therapist knowledge patterns affect patient satisfaction. The authors concluded the therapist characteristics that affect clinical outcome seem to be internal to the therapist and depend on the patient treated
Shen et al. (2018)	The effects of race and racial concordance on patient-physician communication: a systematic review of the literature	Megan Johnson Shen; mes2050@ med.cornell.edu	USA	40	Review studies that examine main effect of patient race and interaction effect of physician and patient race on observational and patient-reported patient-physician communication. Examine whether effects differ by clinical setting	Systematic review	1.) Peer-reviewed, quantitative studies; 2.) Included a patient population sample, compared black to white patients, or racial concordance to discordance; 2.) Assessed patient-physician communication within a medical setting; 3.) Measured communication through audio/video recordings, observation, or patient surveys; and 4.) Published between 1995–2016	(1) Not in an adult patient/health setting; (2) Non-original data; (3) Did not fit authors'definition of patient-physician communication; (4) No comparative analysis between Black and White patients; (5) Non-U.S. study in English; (6) Post-intervention assessment of communication; and (7) Communication was not a clear dependent variable	The effect of physician–patient racial concordance on communication and whether communication patterns differed by setting	Majority of studies report that black patients experience poorer patient-physician communication and outcomes than white patients. Black patients suffer from lower quality and satisfaction, more negative talk, and shorter visits. Race and racial concordance had less of an impact on communication on HIV/AIDS settings. Racial concordance was associated with better outcomes in all areas except quality of communication. Racial concordance acts as a consistent predictor for better patient-physician outcomes
Shin et al. (2005)	A meta-analytic review of racial-ethnic matching for African American and Caucasian American clients and clinicians	Sung-Man Shin; sshin@handong.edu	USA	10	Use meta-analysis to resolve differences in contradictory findings from studies of the effect of physician–patient racial/ethnic concordance	Meta-analysis	1.) Studies involving mental health or substance abuse services; 2.) Studies involving psychotherapy; 3.) Experimental or quasi-experimental designs; 4.) Included findings for Black clients; and 5.) Reported on attrition, total number of sessions attended, and psychosocial functioning	1.) Did not measure the effects of racial-ethnic matching for African-American and Caucasian clients and clinicians; 2.) Did not contain sufficient quantitative outcome data; and 3.) Duplicative data	1.) Patient attrition; 2.) Total number of sessions; attended; and 3.) Psychosocial functioning	No statistically significant difference in retention, number of sessions attended, and posttreatment functioning status was found in regard to racial-matching for both African Americans and Caucasian Americans
Whaley (2001)	Cultural mistrust and mental health services for African Americans: a review and meta-analysis	Arthur L. Whaley; alw2@columbia.edu	USA	22	a) Conduct a meta-analysis of the correlations between African Americans’ cultural mistrust and their attitudes and behaviors related to mental health services use, comparing them to the correlations between cultural mistrust and measures relevant to other psychosocial domains, and (b) to test some methodological hypothesis about the CMI, the most popular measure of cultural mistrust	Meta-analysis	1.) Empirical studies and Dissertations; 2.) Addressed cultural mistrust; 3.) Focused on African American patients or populations	1.) Non-empirical studies; 2.) Examined the relationship between cultural mistrust and racial identity; 3.) Duplicative data; 4.) Studies where the correlations between cultural mistrust scores and attitudes toward psychotherapy for Black respondents could not be determined	1.) Assess the homogeneity of the effect sizes for the included studies; and 2.) Determine the moderating effects of certain study characteristics	The meta-analysis of the literature produced a medium effect size. The results also indicated that the effect size did not differ for counseling and psychotherapy studies compared with other types of studies. Studies that used the complete Cultural Mistrust Inventory were found to be significant. A significant negative association was found between age and cultural mistrust (i.e., younger people were more mistrustful than older). Finally, effect sizes based on multivariate results were much smaller in comparison to studies that used t-tests or zero-order correlations

**Table 3:** Systematic review references matched to primary studies

Publications	Systematic review references
Cooper LA, Roter DL, Johnson RL, Ford DE, Steinwachs DM, Powe NR. Patient-centered communication, ratings of care, and concordance of patient and physician race. Ann Intern Med. 2003;139(11):907–915. https://doi.org/10.7326/0003-4819-139-11-200312020-00009	Shen et al. (2018) Miller et al. (2024)
Gordon HS, Street RL Jr, Kelly PA, Souchek J, Wray NP. Physician–patient communication following invasive procedures: an analysis of post-angiogram consultations. Soc Sci Med. 2005;61(5):1015–1025. https://doi.org/10.1016/j.socscimed.2004.12.021	
Schnittker J, Liang K. The promise and limits of racial/ethnic concordance in physician–patient interaction. *J Health Polit Policy Law*. 2006;31(4):811–838. https://doi.org/10.1215/03616878-2006-004	
Saha S, Komaromy M, Koepsell TD, Bindman AB. Patient-physician racial concordance and the perceived quality and use of health care. *Arch Intern Med*. 1999;159(9):997–1004. https://doi.org/10.1001/archinte.159.9.997	
Stepanikova I, Zhang Q, Wieland D, Eleazer GP, Stewart T. Non-verbal communication between primary care physicians and older patients: how does race matter?. *J Gen Intern Med*. 2012;27(5):576–581. https://doi.org/10.1007/s11606-011-1934-z	
Jerant A, Bertakis KD, Fenton JJ, Tancredi DJ, Franks P. Patient-provider sex and race/ethnicity concordance: a national study of healthcare and outcomes. *Med Care*. 2011;49(11):1012–1020. https://doi.org/10.1097/MLR.0b013e31823688ee	
Street RL Jr, O'Malley KJ, Cooper LA, Haidet P. Understanding concordance in patient-physician relationships: personal and ethnic dimensions of shared identity. *Ann Fam Med*. 2008;6(3):198–205. https://doi.org/10.1370/afm.821	
Martin KD, Roter DL, Beach MC, Carson KA, Cooper LA. Physician communication behaviors and trust among black and white patients with hypertension. *Med Care*. 2013;51(2):151–157. https://doi.org/10.1097/MLR.0b013e31827632a2	
Sweeney CF, Zinner D, Rust G, Fryer GE. Race/ethnicity and health care communication: does patient-provider concordance matter?. *Med Care*. 2016;54(11):1005–1009. https://doi.org/10.1097/MLR.0000000000000578	
Gordon HS, Street RL Jr, Sharf BF, Souchek J. Racial differences in doctors'information-giving and patients'participation. *Cancer*. 2006;107(6):1313–1320. https://doi.org/10.1002/cncr.22122	
Ghods BK, Roter DL, Ford DE, Larson S, Arbelaez JJ, Cooper LA. Patient-physician communication in the primary care visits of African Americans and whites with depression. *J Gen Intern Med*. 2008;23(5):600–606. https://doi.org/10.1007/s11606-008-0539-7	
Johnson RL, Roter D, Powe NR, Cooper LA. Patient race/ethnicity and quality of patient-physician communication during medical visits. *Am J Public Health*. 2004;94(12):2084–2090. https://doi.org/10.2105/ajph.94.12.2084	
Street RL Jr, Gordon H, Haidet P. Physicians'communication and perceptions of patients: is it how they look, how they talk, or is it just the doctor?. *Soc Sci Med*. 2007;65(3):586–598. https://doi.org/10.1016/j.socscimed.2007.03.036	
Chinman MJ, Rosenheck RA, Lam JA. Client-case manager racial matching in a program for homeless persons with serious mental illness. Psychiatr Serv. 2000;51(10):1265–1272. https://doi.org/10.1176/appi.ps.51.10.1265	Shin, et al. (2005) Cabral and Smith (2011)
Gamst, G., Dana, R. H., Der-Karabetian, A., & Kramer, T. (2004). Ethnic match and treatment outcomes for child and adolescent mental health center clients. Journal of Counseling & Development, 82(4), 457–465. https://doi.org/10.1002/j.1556-6678.2004.tb00334.x	
Pierce, K. J. (1999). The effect of matching client and case manager on gender and race on outcomes of case management with the homeless mentally ill. Dissertation Abstracts International: Section B: The Sciences and Engineering, 60(6-B), 2956	
Reams, L.F. (1998)African-American families in psychotherapy: the effects of therapist race (Unpublished doctoral dissertation)	
Sterling RC, Gottheil E, Weinstein SP, Serota R. The effect of therapist/patient race- and sex-matching in individual treatment. Addiction. 2001;96(7):1015–1022. https://doi.org/10.1046/j.1360-0443.2001.967101511.x	
Yeh, M., Takeuchi, D. T., & Sue, S. (1994). Asian-American children treated in the mental health system: a comparison of parallel and mainstream outpatient service centers. Journal of Clinical Child Psychology, 23(1), 5–12. https://doi.org/10.1207/s15374424jccp2301_2	Griner and Smith (2006) Cabral and Smith (2011)
Mathews CA, Glidden D, Murray S, Forster P, Hargreaves WA. The effect on treatment outcomes of assigning patients to ethnically focused inpatient psychiatric units. Psychiatr Serv. 2002;53(7):830–835. https://doi.org/10.1176/ps.53.7.830	
Flaskerud JH, Hu LT. Participation in and outcome of treatment for major depression among low income Asian-Americans. Psychiatry Res. 1994;53(3):289–300. https://doi.org/10.1016/0165-1781(94)90056-6	
Florentine, R., & Hillhouse, M. P. (1999). Drug treatment effectiveness and client-counselor empathy: exploring the effects of gender and ethnic congruency. Journal of Drug Issues, 29(1), 59–74. https://doi.org/10.1177/002204269902900104	Maramba et al. (2002) Griner and Smith (2006)
Sue S, Fujino DC, Hu LT, Takeuchi DT, Zane NW. Community mental health services for ethnic minority groups: a test of the cultural responsiveness hypothesis. J Consult Clin Psychol. 1991;59(4):533–540. https://doi.org/10.1037//0022-006x.59.4.533	
Wintersteen, M. B., Mensinger, J. L., & Diamond, G. S. (2005). Do gender and racial differences between patient and therapist affect therapeutic alliance and treatment retention in adolescents? Professional Psychology: Research and Practice, 36(4), 400–408. https://doi.org/10.1037/0735-7028.36.4.400	DeHaan et al. (2018) Carballeira-Carrera et al. (2011)
Halliday-Boykins CA, Schoenwald SK, Letourneau EJ. Caregiver-therapist ethnic similarity predicts youth outcomes from an empirically based treatment. J Consult Clin Psychol. 2005;73(5):808–818. https://doi.org/10.1037/0022-006X.73.5.808	
Blank MB, Tetrick FL 3rd, Brinkley DF, Smith HO, Doheny V. Racial matching and service utilization among seriously mentally ill consumers in the rural South. Community Ment Health J. 1994;30(3):271–281. https://doi.org/10.1007/BF02188887	Maramba et al. (2002) Shin et al. (2005)
Yeh, M., Eastman, K., & Cheung, M. K. (1994). Children and adolescents in community health centers: does the ethnicity or the language of the therapist matter? Journal of Community Psychology, 22(2), 153–163. https://doi.org/10.1002/1520-6629(199404)22:2<153::AID-JCOP2290220210>3.0.CO;2-R	Maramba, (2002) DeHaan, (2018) Carballeira-Carrera, (2011)
Sterling RC, Gottheil E, Weinstein SP, Serota R. Therapist/patient race and sex matching: treatment retention and 9-month follow-up outcome. Addiction. 1998;93(7):1043–1050. https://doi.org/10.1046/j.1360-0443.1998.93710439.x	Shin et al. (2005) Carballeira- Carrera et al. (2011) Maramba et al. (2002) Sanchez-Bahillo et al. (2014)

### Quality of Systematic Reviews

Using the AMSTAR-2 rating methodology, three reviews were rated as “High” (no or one non-critical weaknesses) [30, 32, 38], one was rated as “Moderate” (more than one non-critical weakness) [9], five were rated as “Low” (one critical flaw with or without non-critical weaknesses) [33–37], and one was rated as “Critically Low” (more than one critical flaw with or without non-critical weaknesses) [31]. Table 4 provides the ratings and overall critical appraisal scores for the included reviews. In terms of critical weaknesses, nine reviews made no mention of an established protocol prior to beginning the study. Similarly, eight out of 10 reviews did not provide a list of excluded studies or assess for risk of bias in each individual study.

**Table 4:**
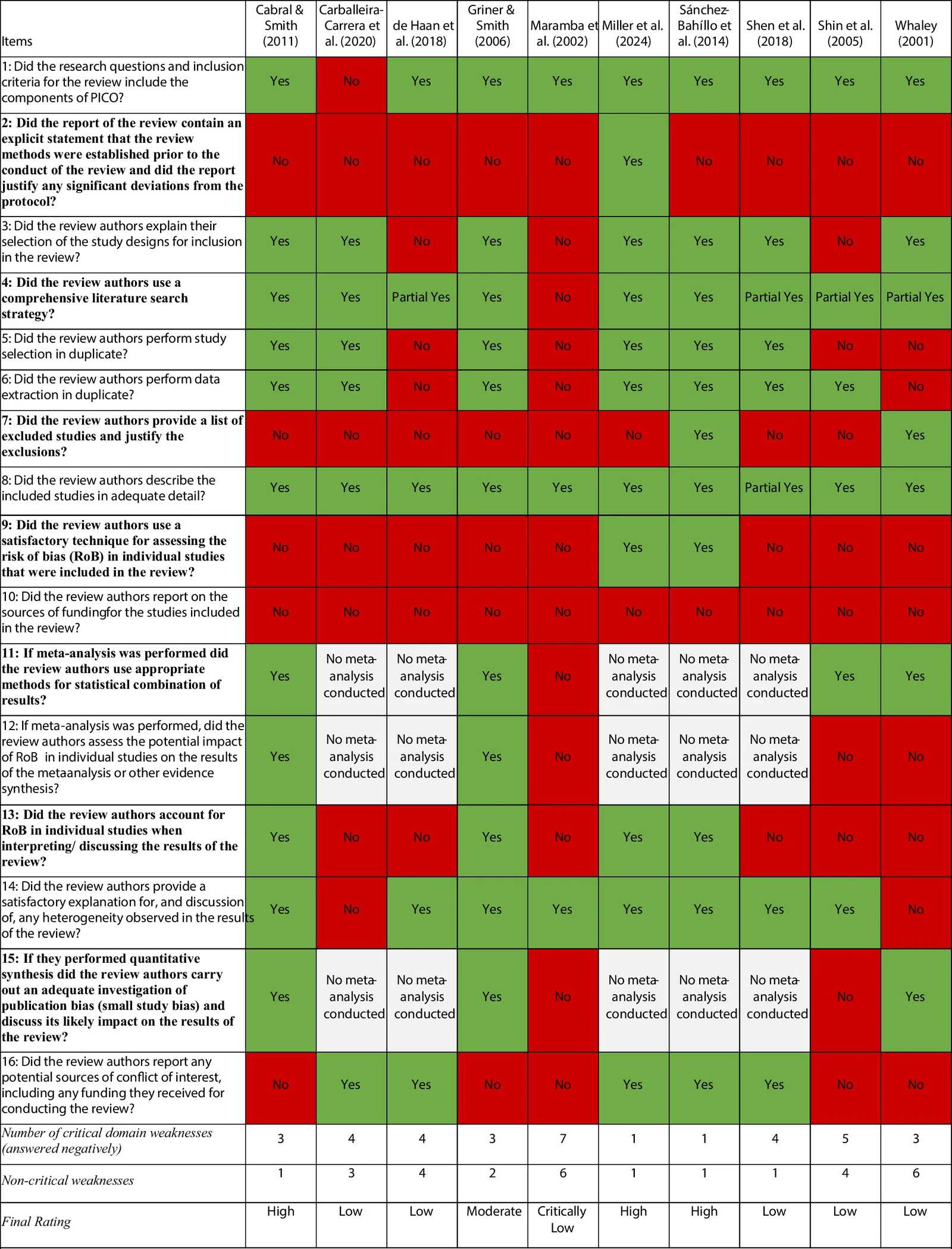
Critical Appraisal Ratings of Included Studies

Bolded items represent critical domains

### Review Focus Areas and Findings

The most frequently studied outcome in the included reviews were related to patient-level outcomes such as dropout rates and satisfaction with services [30, 31, 34, 37, 38]. Additionally, three articles addressed the relevance of culture in the provision of mental health services [9, 35, 36]. Lastly, two articles discussed the effect of physician–patient racial and ethnic concordance on communication during patient encounters [32, 33].

While the collective results of the reviews were heterogenous, there were commonalities identified. Black clients typically experience poorer mental health outcomes in comparison to their counterparts and racial/ethnic matching tends to have a greater effect for this group [30, 33–35, 37, 38]. For example, Cabral and Smith mostly found no significant effects for ethnic matching in terms of treatment outcomes. However, they did discover that Black clients showed the greatest effect sizes in terms of preferences, perceptions, and outcomes when ethnically matched with a therapist [30]. Shen et al. found similar findings when studying the effects of racial concordance on physician–patient communication. In their systematic review, they found that despite heterogenous results, concordance was a dependable predictor for better communication outcomes for Black patients [33].

Additionally, adolescents and young adults from racial and ethnically marginalized groups fared better with concordant providers. Sánchez et al. found strong effects for concordance with adolescents from racial and ethnic marginalized groups attending family therapy [38]. de Haan et al. found that racial and ethnic concordance decreased dropout rates among adolescents in therapy [37]. Whaley also noted a significant relationship between age and cultural mistrust, specifically that younger adults were more distrustful of therapists than older adults [35]. He advises cultural mistrust among younger adults may reflect a stronger preference for racially, ethnically, and/or culturally concordant providers [35].

Furthermore, a few articles discussed the importance of understanding and integrating cultural perspectives into therapeutic techniques and interventions [9, 35, 36]. Griner and Smith found a moderately strong effect for culturally adapted interventions in their systematic review. Specifically, group therapy with a targeted group was four times more effective than those with a mixed group in terms of race and ethnicity. Group interventions that were conducted in participants’ native language were twice as effective [9]. Whaley commented on the importance of culture in his study of the effect of cultural mistrust on mental health services for Black patients [35]. He found the effects of cultural mistrust extended beyond mental health settings and affected behaviors and attitudes in other settings as well [35]. Thus, attention must be paid to the broader cultural milieu and its’ effect on the lived experience for Black patients when providing mental health services [35].

## Discussion

In light of the findings from this review, there are a few key points and recommendations to be made about this type of research. Culture and concordance cannot be distilled to a training, therapeutic technique, or a single factor. Currently, empirical research does not support specific trainings, guidelines, or recruitment best practices for cultural competency [38]. Cultural competency training may not be seen as a cure all to fill the gap left by a lack of racially or ethnically concordant providers. To this point, a few reviews discussed the shallow and overly broad conceptualization of race and ethnicity and cultural matching in the current literature [32, 37, 38]. If culture is thought of in overly broad terms and views racial/ethnic groups as a monolith, then the modalities of cultural competency training will lack nuance and miss the complexity of self-identification and acculturation.

For example, a few reviews highlighted differences in physician preferences based on age [35, 37, 38]. Sánchez-Bahíllo et al. and Whaley posit that identity formation may play a role in this effect. Adolescents who are less acculturated and are still developing a sense of self may prefer a mental health provider that more closely aligns with their emerging identity [35, 38]. While the exact nature of this phenomenon and is not fully understood, all three articles consistently highlight the multifaceted nature of factors influencing outcomes. These include socioeconomic status, therapists’ adherence to therapeutic techniques, therapists’ cultural awareness, and shared racial, ethnic, and cultural backgrounds. In general, these findings suggest that developmental stages could influence preferences for concordant physicians and some mental health outcomes. To deliver high-quality care, physicians need to consider both cultural factors and the patient’s stage of life.

Overall, there is some evidence that racial and ethnic concordance for certain groups can assist in building rapport in the therapist-patient relationship. While positive treatment outcomes are not guaranteed, concordance may be considered a necessary but non-sufficient component of building a therapeutic alliance [34]. Other variables such as shared values, shared personal characteristics, use of patient-centered communication styles, cultural awareness, and cultural commitment have all been found to be moderators in the relationship between therapeutic alliances and mental health outcomes [34, 39, 40]. And, these factors may have greater or lesser effects based on client characteristics such as age, acculturation, or socioeconomic status. The research conducted thus far has often focused on a single component of concordance, such as race or gender, or has focused on medical specialties or outcomes other than mental health. For example, a study conducted by Thornton et al. used a composite measure of shared social characteristics (e.g., race, gender, age, education) [41]. In their results, they found a greater likelihood of positive perceptions among physician–patient dyads with high social concordance [41]. Future research should focus on multiple measures of concordance, the intersection between them, and investigate mediators and moderators to therapeutic outcomes. This would deepen the understanding of concordance, the factors that facilitate better therapeutic alliances, and recommendations about best practices for the use of social matching in mental health.

## Strengths and Limitations

There are a few limitations to this study that must be acknowledged. First, low critical appraisal scores can reduce confidence in the results of each study. By extension, the low quality of the reviews may have influenced the results presented in this review. Another limitation of this study was the inclusion of English-only peer-reviewed reviews. There may be systematic reviews in other languages that could have provided information on physician–patient racial and ethnic concordance. The primary strength of this study is the synthesis of 24 years of research on this topic across 72 studies. Further, the use of AMSTAR 2 tool allows for uniform and replicable critical appraisal articles as well as use of prescriptive methods for conducting a review of reviews.

## Conclusion

Although there were heterogenous results within the included reviews, many found Black clients experienced greater health disparities in comparison to their counterparts. Furthermore, racial/ethnic matching tends to have a greater impact on this group [30, 33–35, 37, 38]. This finding is also supported by discussions found in the literature related to cultural mistrust and cultural competency training. Currently, empirical research does not support specific trainings, guidelines, or recruitment best practices for cultural competency [38]. Given that racial, ethnic, and cultural identity are nuanced and complex, it is not reasonable to believe a simple training can prepare therapists to meaningfully consider culture in their work. Moreover, the traits that enhance the therapeutic relationship often extend beyond race and ethnicity. Future research in this area should focus on the multiple measures of concordance, the intersection between them, and investigate mediators and moderators to therapeutic outcomes. This would strengthen what is already known about concordance and add to the body of literature on mental health for historically marginalized or minoritized populations.

## Data Availability

All of the articles used in this review of reviews are publicly available and accessible using searchable citation databases.

## Declarations

### Disclaimer

The contents of this publication are the sole responsibility of the authors and do not necessarily reflect the views, assertions, opinions, or policies of the Uniformed Services University of the Health Sciences (USUHS), the Henry M. Jackson Foundation for the Advancement of Military Medicine, Inc. (HJF), the Department of Defense (DoD), or the Departments of the Army, Navy, or Air Force. Mention of trade names, commercial products, or organizations does not imply endorsement by the US Government.

## References

[1] Harrison DK. Race as a counselor-client variable in counseling and psychotherapy: a review of the research. Couns Psychol. 1975;5(1):124–33.

[2] Sue S. Psychotherapeutic services for ethnic minorities: two decades of research findings. Am Psychol. 1988;43(4):301–8. 3289427 10.1037//0003-066x.43.4.301

[3] Meghani SH, et al. Patient-provider race-concordance: does it matter in improving minority patients’ health outcomes? Ethn Health. 2009;14(1):107–30. 19012091 10.1080/13557850802227031 PMC3209820

[4] Stevens GD, Mistry R, Halfon N. Caution urged in concluding disparities mitigated by increase in AA docs. J Natl Med Assoc. 2003;95(8):755–6. 12934877 PMC2594572

[5] Takeuchi DT, Sue S, Yeh M. Return rates and outcomes from ethnicity-specific mental health programs in Los Angeles. Am J Public Health. 1995;85(5):638–43. 7733422 10.2105/ajph.85.5.638 PMC1615442

[6] Mujica C, et al. Association between patient-provider racial and ethnic concordance and patient-centered communication in outpatient mental health clinics. J Psychother Integr. 2020;30(3):423–39.

[7] Brach, C. and I. Fraserirector, *Can cultural competency reduce racial and ethnic health disparities? A review and conceptual model.* Medical care research and review, 2000;57(1_suppl): p. 181–217. 10.1177/1077558700057001S09 PMC5091811 11092163

[8] Beach MC, et al. Cultural competence: a systematic review of health care provider educational interventions. Med Care. 2005;43(4):356–73. 15778639 10.1097/01.mlr.0000156861.58905.96 PMC3137284

[9] Griner, D. and T.B. Smith, *Culturally adapted mental health interventions: A meta-analytic review.* Psychotherapy (Chicago, Ill.), 2006;43(4): p. 531–548. 10.1037/0033-3204.43.4.531 22122142

[10] Lie DA, et al. Does cultural competency training of health professionals improve patient outcomes? A systematic review and proposed algorithm for future research. J Gen Intern Med. 2011;26(3):317–25. 20953728 10.1007/s11606-010-1529-0 PMC3043186

[11] Truong M, Paradies Y, Priest N. Interventions to improve cultural competency in healthcare: a systematic review of reviews. BMC Health Serv Res. 2014;14(1):99. 24589335 10.1186/1472-6963-14-99 PMC3946184

[12] Sass B, et al. Enhancing pathways to care for black and minority ethnic populations: a systematic review. Int Rev Psychiatry. 2009;21(5):430–8. 20374158 10.1080/09540260802204121

[13] Van Keer RV, et al. Dealing with cultural diversity during the process of communication and decision-making in the ICU: A literature review. Crit Care. 2014;18:S9.

[14] Bhui, K., et al., *Interventions designed to improve therapeutic communications between black and minority ethnic people and professionals working in psychiatric services: a systematic review of the evidence for their effectiveness.* Health Technol Assess, 2015;19(31): p. vii-xxiv, 1–173. 10.3310/hta19310 PMC4780955 25921977

[15] Lu W, et al. Barriers and facilitators for mental health service use among racial/ethnic minority adolescents: a systematic review of literature. Front Public Health. 2021;9: 641605. 33763401 10.3389/fpubh.2021.641605 PMC7982679

[16] Grant MJ, Booth A. A typology of reviews: an analysis of 14 review types and associated methodologies. Health Info Libr J. 2009;26(2):91–108. 19490148 10.1111/j.1471-1842.2009.00848.x

[17] Higgins JPT, T.J., Chandler J, Cumpston M, Li T, Page MJ, Welch VA (eds). *Cochrane handbook for systematic reviews of interventions version 6.5 (updated August 2024)*. 2024; Available from: www.training.cochrane.org/handbook.

[18] Aromataris E, et al. Summarizing systematic reviews: methodological development, conduct and reporting of an umbrella review approach. Int J Evid Based Healthcare. 2015;13(3):132–40. 10.1097/XEB.0000000000000055 26360830

[19] Bruyère Research Institute, *A MeaSurement Tool to Assess systematic Reviews (AMSTAR-2) Checklist* 2023.

[20] Shea BJ, et al. AMSTAR 2: A critical appraisal tool for systematic reviews that include randomised or non-randomised studies of healthcare interventions, or both. BMJ. 2017;358: j4008. 28935701 10.1136/bmj.j4008 PMC5833365

[21] Jani M, et al. Investigation of idiopathic inflammatory myopathy for shared genetic risk factors with other autoimmune diseases: results from the European myositis network. The Lancet. 2013;381:S56.

[22] Jones, C., et al., *Virtual trauma-focused therapy for military members, veterans, and public safety personnel with posttraumatic stress injury: Systematic scoping review.* JMIR mHealth and uHealth, 2020; 8(9). 10.2196/22079 PMC7536597 32955456

[23] Kenny DA, West TV. Similarity and agreement in self-and other perception: a meta-analysis. Pers Soc Psychol Rev. 2010;14(2):196–213. 20142435 10.1177/1088868309353414

[24] Lassell RKF, et al. Hospice interventions for persons living with dementia, family members and clinicians: a systematic review. J Am Geriatr Soc. 2022;70(7):2134–45. 35441699 10.1111/jgs.17802 PMC9283206

[25] Linton JM, Feudtner C. What accounts for differences or disparities in pediatric palliative and end-of-life care? A systematic review focusing on possible multilevel mechanisms. Pediatrics. 2008;122(3):574–82. 18762528 10.1542/peds.2007-3042

[26] Peters J, et al. The impact of race/ethnicity on the outcomes of burn patients: a systematic review of the literature. J Burn Care Res. 2022;43(2):323–35. 34520543 10.1093/jbcr/irab174

[27] Zhao C, et al. Race, gender, and language concordance in the care of surgical patients: a systematic review. Surgery. 2019;166(5):785–92. 31375322 10.1016/j.surg.2019.06.012

[28] Miller, A.N., et al., *The relationship of ethnic, racial, and cultural concordance to physician-patient communication: a mixed-methods systematic review protocol.* Health Commun, 2022: p. 1–7. 10.1080/10410236.2022.2070449 35502565

[29] Vo T, Canty L. Global mental health experiences of single mothers: a mixed methods research synthesis. J Adv Nurs. 2023;79(1):68–82. 36226874 10.1111/jan.15461

[30] Cabral RR, Smith TB. Racial/ethnic matching of clients and therapists in mental health services: a meta-analytic review of preferences, perceptions, and outcomes. J Couns Psychol. 2011;58(4):537–54. 21875181 10.1037/a0025266

[31] Maramba, G.G. and G.C. Nagayama Hall, *Meta-analyses of ethnic match as a predictor of dropout, utilization, and level of functioning.* Cultural diversity & ethnic minority psychology, 2002;8(3): p. 290–297. 10.1037/1099-9809.8.3.290 12143105

[32] Miller AN, et al. The relationship of race/ethnicity concordance to physician-patient communication: a mixed-methods systematic review. Health Commun. 2024;39(8):1543–57. 37338139 10.1080/10410236.2023.2223402

[33] Shen MJ, et al. The effects of race and racial concordance on patient-physician communication: a systematic review of the literature. J Racial Ethn Health Disparities. 2018;5(1):117–40. 28275996 10.1007/s40615-017-0350-4 PMC5591056

[34] Shin S-M, et al. A meta-analytic review of racial-ethnic matching for African American and Caucasian American clients and clinicians. J Couns Psychol. 2005;52(1):45–56.

[35] Whaley AL. Cultural mistrust and mental health services for African Americans: a review and meta-analysis. Couns Psychol. 2001;29(4):513–31.

[36] Carballeira Carrera, L., et al., *Clinical approaches to cultural diversity in mental health care and specificities of French transcultural consultations: a scoping review.* Frontiers in Psychiatry 2020;11. 10.3389/fpsyt.2020.579147 PMC7652727 33192718

[37] de Haan AM, et al. A review of mental health treatment dropout by ethnic minority youth. Transcult Psychiatry. 2018;55(1):3–30. 29035137 10.1177/1363461517731702

[38] Sánchez-Bahíllo Á, et al. Therapist characteristics that predict the outcome of multipatient psychotherapy: systematic review of empirical studies. J Psychiatr Res. 2014;53:149–56. 24582149 10.1016/j.jpsychires.2014.01.016

[39] Huang CY, Zane N. Cultural influences in mental health treatment. Curr Opin Psychol. 2016;8:131–6. 29506788 10.1016/j.copsyc.2015.10.009 PMC9528809

[40] Bowman, R.C., *The effects of client therapist racial and ethnic matching: a meta analytic review of empirical research*. 2022, ProQuest Information & Learning.

[41] Thornton RL, et al. Patient-physician social concordance, medical visit communication and patients’ perceptions of health care quality. Patient Educ Couns. 2011;85(3):e201–8. 21840150 10.1016/j.pec.2011.07.015 PMC3217162

